# Supplementary material for: On the coherence of model-based dose-finding designs for drug combination trials
Source: PLoS One. 2020 Nov 30;15(11):e0242561. doi: 10.1371/journal.pone.0242561 (PMC7703981; doi:10.1371/journal.pone.0242561)
Supplement: S1 File — (PDF) [file pone.0242561.s001.pdf]

# Supplementary Materials for "On the coherence of model-based dose-finding designs for drug combination trials"

Yeonhee Park[1] and Suyu Liu[2,*]

[1] Department of Biostatistics and Medical Informatics, University of Wisconsin-Madison

[2] Department of Biostatistics, The University of Texas MD Anderson Cancer Center

[*] email: syliu@mdanserson.org

## Appendix A: Proofs of theoretical results in Materials and methods

**Proof of Theorem 1** Suppose that the $n$th patient is treated at $X_n = (j', k')$ for any $n$. By the mean value theorem, we have

$$F(u_{j'}, v_{k'}, \hat{\boldsymbol{\theta}}_n) - F(u_{j'}, v_{k'}, \hat{\boldsymbol{\theta}}_{n-1}) = \sum_{t=1}^{p} (\hat{\theta}_{n,t} - \hat{\theta}_{n-1,t}) \frac{\partial F(u_{j'}, v_{k'}, \tilde{\boldsymbol{\theta}})}{\partial \theta_t},$$

where $\tilde{\boldsymbol{\theta}} = \hat{\boldsymbol{\theta}}_n + \alpha(\hat{\boldsymbol{\theta}}_{n-1} - \hat{\boldsymbol{\theta}}_n)$ for some $\alpha \in [0, 1]$. In case of $Y_n = 1$, from condition A, we have $F(u_{j'}, v_{k'}, \hat{\boldsymbol{\theta}}_n) \geq F(u_{j'}, v_{k'}, \hat{\boldsymbol{\theta}}_{n-1})$, implying that $\Pr\{X_{n+1} \in \mathcal{E}_n | Y_n = 1\} = 0$. Also, in case of $Y_n = 0$, from condition A, we have $F(u_{j'}, v_{k'}, \hat{\boldsymbol{\theta}}_n) \leq F(u_{j'}, v_{k'}, \hat{\boldsymbol{\theta}}_{n-1})$, implying that $\Pr\{X_{n+1} \in \mathcal{D}_n | Y_n = 0\} = 0$. $\square$

**Proof of Lemma 2** Let $\hat{\boldsymbol{\theta}}_n = (\hat{\theta}_{n,1}, \ldots, \hat{\theta}_{n,p})^\top$ be a posterior mean of $\boldsymbol{\theta}$ with the likelihood function $\mathcal{L}_n(\boldsymbol{\theta})$ based on the accumulating data. We claim that (1) For a uniformly nondecreasing function $F$, $Y_n = 1$ and $\hat{\theta}_{n,t} \geq \hat{\theta}_{n-1,t}$ for all $t$. (2) For a uniformly nondecreasing function $F$, $Y_n = 0$ and $\hat{\theta}_{n,t} \leq \hat{\theta}_{n-1,t}$ for all $t$. (3) For a uniformly nonincreasing function $F$, $Y_n = 1$ and $\hat{\theta}_{n,t} \leq \hat{\theta}_{n-1,t}$ for all $t$. (4) For a uniformly nonincreasing function $F$, $Y_n = 0$ and $\hat{\theta}_{n,t} \geq \hat{\theta}_{n-1,t}$ for all $t$. Since the same logic is used to prove the above four statements, we show only the last statement here.

Suppose that $F$ is nonincreasing in $\theta_t$ for all $t$ and $Y_n = 0$. Since $\hat{\boldsymbol{\theta}}_n$ denotes the posterior mean of $\boldsymbol{\theta}$ with the likelihood function $\mathcal{L}_n(\boldsymbol{\theta}) = \mathcal{L}_{n-1}(\boldsymbol{\theta})\{1 - F(u', v', \boldsymbol{\theta})\}$, we have

$$\hat{\boldsymbol{\theta}}_n - \hat{\boldsymbol{\theta}}_{n-1} = \frac{\int \boldsymbol{\theta} f(\boldsymbol{\theta})\mathcal{L}_n(\boldsymbol{\theta})\, d\boldsymbol{\theta}}{\int f(\boldsymbol{\theta})\mathcal{L}_n(\boldsymbol{\theta})\, d\boldsymbol{\theta}} - \frac{\int \boldsymbol{\theta} f(\boldsymbol{\theta})\mathcal{L}_{n-1}(\boldsymbol{\theta})\, d\boldsymbol{\theta}}{\int f(\boldsymbol{\theta})\mathcal{L}_{n-1}(\boldsymbol{\theta})\, d\boldsymbol{\theta}} \equiv M_1^{-1}\boldsymbol{M}_2,$$

where

$$M_1 = \int\int f(\boldsymbol{\phi})f(\boldsymbol{\psi})\mathcal{L}_n(\boldsymbol{\phi})\mathcal{L}_{n-1}(\boldsymbol{\psi})\, d\boldsymbol{\phi}d\boldsymbol{\psi}$$

and

$$\boldsymbol{M}_2 = \int\int \boldsymbol{\phi} f(\boldsymbol{\phi})f(\boldsymbol{\psi})\mathcal{L}_{n-1}(\boldsymbol{\phi})\mathcal{L}_{n-1}(\boldsymbol{\psi})\big\{F(u', v', \boldsymbol{\psi}) - F(u', v', \boldsymbol{\phi})\big\}\, d\boldsymbol{\phi}d\boldsymbol{\psi}.$$

We note that

$$\boldsymbol{M}_2 = -\int\int \boldsymbol{\psi} f(\boldsymbol{\phi})f(\boldsymbol{\psi})\mathcal{L}_{n-1}(\boldsymbol{\phi})\mathcal{L}_{n-1}(\boldsymbol{\psi})\big\{F(u', v', \boldsymbol{\psi}) - F(u', v', \boldsymbol{\phi})\big\}\, d\boldsymbol{\phi}d\boldsymbol{\psi}.$$

From () and (), we have

$$2\boldsymbol{M}_2 = \int\int (\boldsymbol{\phi} - \boldsymbol{\psi})\big\{F(u', v', \boldsymbol{\psi}) - F(u', v', \boldsymbol{\phi})\big\} f(\boldsymbol{\phi})f(\boldsymbol{\psi})\mathcal{L}_n(\boldsymbol{\phi})\mathcal{L}_n(\boldsymbol{\psi})\, d\boldsymbol{\phi}d\boldsymbol{\psi}.$$

Let $\boldsymbol{\phi} = (\phi_1, \ldots, \phi_p)^\top$ and $\boldsymbol{\psi} = (\psi_1, \ldots, \psi_p)^\top$. Then, for each $t$,

$$(\phi_t - \psi_t)\big\{F(u', v', \boldsymbol{\psi}) - F(u', v', \boldsymbol{\phi})\big\} = -(\phi_t - \psi_t)^2 \frac{\partial F(u', v', \boldsymbol{\theta}^*)}{\partial \theta_t} \geq 0,$$

where $\boldsymbol{\theta}^* = \boldsymbol{\phi} + \alpha(\boldsymbol{\psi} - \boldsymbol{\phi})$ for some $\alpha \in [0, 1]$, because $F$ is nonincreasing in $\theta_t$. Thus, each element of $\mathbf{M}_2$ is nonnegative and $\hat{\theta}_{n,t} \geq \hat{\theta}_{n-1,t}$ for any $t$. □

In order to prove Theorem 4 and Theorem 5, we need the following result, which directly induces weak coherence and is used to show strong coherence.

**Lemma 1** *Let $\mathcal{E}_{n,\hat{F}}$ and $\mathcal{D}_{n,\hat{F}}$ denote the set of candidate dose levels for escalation and*

*de-escalation, respectively, for the* $(n+1)$*th patient determined by the model-based toxicity estimate* $F_{j',k'}(\hat{\boldsymbol{\theta}}_n)$*. If condition A or the uniform monotonicity condition holds, then* $\Pr(X_{n+1} \in \mathcal{E}_{n,\hat{F}}|Y_n = 1) = 0$ *and* $\Pr(X_{n+1} \in \mathcal{D}_{n,\hat{F}}|Y_n = 0) = 0$.

Proof. Suppose that the $n$th patient is treated at $X_n = (j', k')$ for any $n$. By the proof of Theorem 1, under condition A, we have $F(u_{j'}, v_{k'}, \hat{\boldsymbol{\theta}}_n) \geq F(u_{j'}, v_{k'}, \hat{\boldsymbol{\theta}}_{n-1})$ when $Y_n = 1$ and $F(u_{j'}, v_{k'}, \hat{\boldsymbol{\theta}}_n) \leq F(u_{j'}, v_{k'}, \hat{\boldsymbol{\theta}}_{n-1})$ when $Y_n = 0$. So, we have $\Pr(X_{n+1} \in \mathcal{E}_{n,\hat{F}}|Y_n = 1) = 0$ and $\Pr(X_{n+1} \in \mathcal{D}_{n,\hat{F}}|Y_n = 0) = 0$. With the same logic, by the proof of Lemma 2, the uniform monotonicity condition gives $\Pr(X_{n+1} \in \mathcal{E}_{n,\hat{F}}|Y_n = 1) = 0$ and $\Pr(X_{n+1} \in \mathcal{D}_{n,\hat{F}}|Y_n = 0) = 0$. $\square$

**Proof of Theorem 4** Suppose that the $n$th patient is treated at $X_n = (j', k')$ for any $n$. Let $\mathcal{E}_{n,\hat{F}} = \{(l,m) : F(u_l, v_m, \hat{\boldsymbol{\theta}}_n) > F(u_{j'}, v_{k'}, \hat{\boldsymbol{\theta}}_n)\}$ and $\mathcal{D}_{n,\hat{F}} = \{(l,m) : F(u_l, v_m, \hat{\boldsymbol{\theta}}_n) < F(u_{j'}, v_{k'}, \hat{\boldsymbol{\theta}}_n)\}$. Then, $\mathcal{E}_{n,\hat{F}}$ and $\mathcal{D}_{n,\hat{F}}$ denote the set of candidate dose levels for escalation and de-escalation, respectively, for the $(n+1)$th patient determined by the model-based toxicity estimate $F_{j',k'}(\hat{\boldsymbol{\theta}}_n)$. Similarly, $\mathcal{E}_{n,p}$ and $\mathcal{D}_{n,p}$ are defined by the true toxicity probability $p_{j',k'}$. By Lemma 1, under either condition A or the uniform monotonicity condition, we have $\Pr(X_{n+1} \in \mathcal{E}_{n,\hat{F}}|Y_n = 1) = 0$ and $\Pr(X_{n+1} \in \mathcal{D}_{n,\hat{F}}|Y_n = 0) = 0$. We now claim that $\mathcal{E}_{n,\hat{F}} = \mathcal{E}_{n,p}$ and $\mathcal{D}_{n,\hat{F}} = \mathcal{D}_{n,p}$ for all $n$. Let $\mathcal{A}_n$ be the set of all possible dose levels for dose assignments from $X_n$. For $(l,m) \in \mathcal{E}_{n,p}$, by conditions B1 and B2, we have $\mathrm{sgn}\{F(u_l, v_m, \boldsymbol{\theta}) - F(u_{j'}, v_{k'}, \boldsymbol{\theta})\} = \mathrm{sgn}(p_{l,m} - p_{j',k'}) = 1$ for any $\boldsymbol{\theta}$. This gives $F(u_l, v_m, \hat{\boldsymbol{\theta}}_n) - F(u_{j'}, v_{k'}, \hat{\boldsymbol{\theta}}_n) > 0$, implying $(l,m) \in \mathcal{E}_{n,\hat{F}}$. So, $\mathcal{E}_{n,p} \subset \mathcal{E}_{n,\hat{F}}$. Similarly, we can show that for any $(l,m) \in \mathcal{E}_{n,\hat{F}}$ , $(l,m) \in \mathcal{E}_{n,p}$, which implies that $\mathcal{E}_{n,\hat{F}} \subset \mathcal{E}_{n,p}$. Thus, $\mathcal{E}_{n,p} = \mathcal{E}_{n,\hat{F}}$. Likewise, we can obtain $\mathcal{D}_{n,p} = \mathcal{D}_{n,\hat{F}}$ for any $n$. Therefore, $\Pr(X_{n+1} \in \mathcal{E}_{n,p}|Y_n = 1) = 0$ and $\Pr(X_{n+1} \in \mathcal{D}_{n,p}|Y_n = 0) = 0$, which means that the D-design is strongly coherent. $\square$

**Proof of Theorem 5** Suppose that the $n$th patient is treated at $X_n = (j', k')$ for any $n$. Similar to the proof of Theorem 4, $\mathcal{E}_{n,\hat{F}}$ and $\mathcal{D}_{n,\hat{F}}$ are defined as the set of candidate dose levels for escalation and de-escalation, respectively, for the $(n+1)$th patient determined by the dose-toxicity model-based toxicity estimate $F_{j',k'}(\hat{\boldsymbol{\theta}}_n)$. By Lemma 1, under condition A, we have $\Pr(X_{n+1} \in \mathcal{E}_{n,\hat{F}} | Y_n = 1) = 0$ and $\Pr(X_{n+1} \in \mathcal{D}_{n,\hat{F}} | Y_n = 0) = 0$. This means that the D-design prohibits dose escalation when the outcome of the recently treated patient shows toxicity and prohibits dose de-escalation when the outcome of the recently treated patient does not show toxicity, where the dose escalation and de-escalation sets are determined by the estimated toxicity probability $F_{j',k'}(\hat{\boldsymbol{\theta}}_n)$. Therefore, the D-design is weakly coherent. With the same logic, by Lemma 1, the uniform monotonicity condition gives the weak coherence of the D-design. □

**Proof of Theorem 6** The proof is trivial. It suffices to check the coherence of the two-stage design at the transition point. Suppose that $Y_M = 0$. Because of the condition, we have $F(X^*_{M+1}, \boldsymbol{\theta}_M) \geq F(X^*_M, \boldsymbol{\theta}_M)$ with probability 1. This means that $X^*_{M+1}$ indicates the dose level for the escalation from the current dose level $X^*_M$ and the dose level for the next patient never de-escalates, i.e., $\Pr\{X_{M+1} \in \mathcal{D}_M | Y_M = 0\} = 0$. Likewise, because $F(X^*_{M+1}, \boldsymbol{\theta}_M) \leq F(X^*_M, \boldsymbol{\theta}_M)$ with probability 1 when $Y_M = 1$, $X^*_{M+1}$ denotes the dose level for de-escalation from the current dose level, and we obtain $\Pr\{X_{M+1} \in \mathcal{E}_M | Y_M = 1\} = 0$. □

## Appendix B: Details of examples in Application

In this appendix, we let $(u_j, v_k)$ denote the dose pair that is used to treat the $n$th patient.

**Example 1a. Generalized CRM model** Braun and Jia (2013) generalized the CRM to a phase I trial with a combination of drugs using the logit link. Let $\eta(x) = \text{logit}(x)$ denote

the logit link. Then, under the generalized CRM model, the dose-toxicity relationship is described by $\text{logit}\{F_{j,k}(\boldsymbol{\theta})\} = \alpha_k + \beta u_j$, where $\boldsymbol{\theta} = (\alpha_1, \ldots, \alpha_K, \beta)$ with $-\infty < \alpha_k < \infty$, $k = 1, \ldots, K$ and $\beta > 0$. Suppose that $Y_n = 0$. We show how condition A is verified.

Step 1. For each $k = 1, \ldots, K$, we take $\boldsymbol{\phi}_k = (\alpha_1, \ldots, \alpha_{k1}, \ldots, \alpha_K)$ and $\boldsymbol{\psi}_k = (\alpha_1, \ldots, \alpha_{k2}, \ldots, \alpha_K)$, for which the elements are fixed except for the $k$th element. Then, $F_{j,k}(\boldsymbol{\phi}_k) - F_{j,k}(\boldsymbol{\psi}_k) = (\alpha_{k1} - \alpha_{k2})/\eta'(\tilde{F}_k)$, where $\tilde{F}_k$ lies between $F_{j,k}(\boldsymbol{\phi}_k)$ and $F_{j,k}(\boldsymbol{\psi}_k)$, and $\eta'(F)$ denotes the derivative of $\eta$ with respect to $F$. Since $\eta'(x) \geq 0$ for $0 < x < 1$, $F_{j,k}(\boldsymbol{\phi}_k) - F_{j,k}(\boldsymbol{\psi}_k) \leq 0$ for $\alpha_{k1} \leq \alpha_{k2}$, i.e., $F$ is nondecreasing in $\alpha_k$. Also, $(\alpha_{k1} - \alpha_{k2})\{F_{j,k}(\boldsymbol{\psi}_k) - F_{j,k}(\boldsymbol{\phi}_k)\} \leq 0$ for any $\alpha_{k1}$ and $\alpha_{k2}$. For each $k = 1, \ldots, K$, let $\hat{\alpha}_{n,k}$ denote the posterior mean of $\alpha_k$ based on the first $n$ patients. Then, by the approach used in the proof of Lemma 2, we have $\hat{\alpha}_{n,k} \leq \hat{\alpha}_{n-1,k}$ for $k = 1, \ldots, K$. Thus, $(\hat{\alpha}_{n,k} - \hat{\alpha}_{n-1,k})\partial F/\partial \alpha_k \leq 0$ for $k = 1, \ldots, K$.

Step 2. Let $\hat{\beta}_n$ denote the posterior mean of $\beta$ based on the first $n$ patients. Take $\boldsymbol{\phi} = (\alpha_1, \ldots, \alpha_K, \beta_1)$ and $\boldsymbol{\psi} = (\alpha_1, \ldots, \alpha_K, \beta_2)$ for any $\beta_1$ and $\beta_2$. Then, $F_{j,k}(\boldsymbol{\phi}) - F_{j,k}(\boldsymbol{\psi}) = (\beta_1 - \beta_2)u_j/\eta'(\tilde{F})$, where $\tilde{F}$ lies between $F_{j,k}(\boldsymbol{\phi})$ and $F_{j,k}(\boldsymbol{\psi})$. For $\beta_1 \leq \beta_2$, the quantity of $F_{j,k}(\boldsymbol{\phi}) - F_{j,k}(\boldsymbol{\psi})$ is $\geq 0$ or $\leq 0$ if $u_j \geq 0$ or $u_j < 0$, respectively. So, $F$ is nondecreasing in $\beta$ if $u_j \geq 0$ and nonincreasing in $\beta$ if $u_j < 0$. Similar to the first step, by using the approach used in the proof of Lemma 2, we have $\hat{\beta}_n \leq \hat{\beta}_{n-1}$ if $u_j \geq 0$ and $\hat{\beta}_n \geq \hat{\beta}_{n-1}$ if $u_j < 0$. Thus, regardless of the sign of $u_j$, we obtain $(\hat{\beta}_n - \hat{\beta}_{n-1})\partial F/\partial \beta \leq 0$.

Therefore, the inequality (3) holds when $Y_n = 0$. We can similarly show that the inequality (3) holds when $Y_n = 1$. □

**Example 1b. Scaled logistic regression model** The scaled logistic regression model is given by $\text{logit}\{F_{j,k}(\boldsymbol{\theta})/\rho\} = \alpha + \beta u_j + \gamma v_k$, where $\boldsymbol{\theta} = (\alpha, \beta, \gamma, \rho)$ with $-\infty < \alpha < \infty$, $\beta > 0, \gamma > 0$ and $0 < \rho \leq 1$. Let $\eta(x) = \text{logit}(x)$. Then, $F_{j,k}(\boldsymbol{\theta}) = \rho\,\eta^{-1}(\alpha + \beta u_j + \gamma v_k)$. As in the logistic regression model, if doses $u_j$ and $v_k$ are nonnegative, it is easy for the scaled

logistic regression model to show that the uniform monotonicity condition holds, while the uniform monotonicity condition does not hold when the doses are not guaranteed, $u_j \geq 0$ and $v_k \geq 0$. Without loss of generality, we suppose that $u_j \geq 0$ and $v_k < 0$. Suppose further that $Y_n = 0$, which implies that $Y_n - p_T < 0$. We show how condition A is checked as follows.

Step 1. Let $\hat{\alpha}_n$, $\hat{\beta}_n$ and $\hat{\gamma}_n$ be posterior mean of $\alpha, \beta$ and $\gamma$, respectively, based on the first $n$ patients. Since $u_j \geq 0$ and $v_k < 0$, $F$ is nondecreasing in $\alpha$ and $\beta$ while $F$ is decreasing in $\gamma$. This implies that (1) for any $\boldsymbol{\phi} = (\alpha_1, \beta, \gamma)$ and $\boldsymbol{\psi} = (\alpha_2, \beta, \gamma)$, we have $(\alpha_1 - \alpha_2)\{F_{j,k}(\boldsymbol{\psi}) - F_{j,k}(\boldsymbol{\phi})\} \leq 0$, which yields $\hat{\alpha}_n \leq \hat{\alpha}_{n-1}$; (2) for any $\boldsymbol{\phi} = (\alpha, \beta_1, \gamma)$ and $\boldsymbol{\psi} = (\alpha, \beta_2, \gamma)$, we have $(\beta_1 - \beta_2)\{F_{j,k}(\boldsymbol{\psi}) - F_{j,k}(\boldsymbol{\phi})\} \leq 0$, which yields $\hat{\beta}_n \leq \hat{\beta}_{n-1}$; (3) for any $\boldsymbol{\phi} = (\alpha, \beta, \gamma_1)$ and $\boldsymbol{\psi} = (\alpha, \beta, \gamma_2)$, we have $(\gamma_1 - \gamma_2)\{F_{j,k}(\boldsymbol{\psi}) - F_{j,k}(\boldsymbol{\phi})\} \geq 0$, which yields $\hat{\gamma}_n \geq \hat{\gamma}_{n-1}$ by the approach used in the proof of Lemma 2. Therefore, $(\hat{\alpha}_n - \hat{\alpha}_{n-1})\partial F/\partial\alpha \leq 0$, $(\hat{\beta}_n - \hat{\beta}_{n-1})\partial F/\partial\beta \leq 0$ and $(\hat{\gamma}_n - \hat{\gamma}_{n-1})\partial F/\partial\gamma \leq 0$.

Step 2. For fixed values of $\alpha, \beta$ and $\gamma$, we take $\boldsymbol{\phi} = (\alpha, \beta, \gamma, \rho_1)$ and $\boldsymbol{\psi} = (\alpha, \beta, \gamma, \rho_2)$ for any $\rho_1$ and $\rho_2$. Then, for $\rho_1 \leq \rho_2$, we have $F_{j,k}(\boldsymbol{\phi}) - F_{j,k}(\boldsymbol{\psi}) = (\rho_1 - \rho_2)\eta^{-1}(\alpha + \beta u_j + \gamma v_k) \leq 0$, because $\eta^{-1}(x) \geq 0$ for all $x$. So, $F$ is nondecreasing in $\rho$ and $(\rho_1 - \rho_2)\{F_{j,k}(\boldsymbol{\psi}) - F_{j,k}(\boldsymbol{\phi})\} \leq 0$ for any $\rho_1$ and $\rho_2$. Let $\hat{\rho}_n$ denote a posterior mean of $\rho$ based on the first $n$ patients. By the approach used in the proof of Lemma 2, $\hat{\rho}_n \leq \hat{\rho}_{n-1}$. Thus, $(\hat{\rho}_n - \hat{\rho}_{n-1})\partial F/\partial\rho \leq 0$.

Therefore, the inequality (3) holds when $Y_n = 0$. We can similarly show that the inequality (3) holds when $Y_n = 1$. □

**Example 2. Change-point model** Let $\boldsymbol{\theta} = (\alpha, \beta, \gamma, w)$, with $-\infty < \alpha < \infty$, $\beta > 0$, $\gamma > 0$ and $\infty < w < \infty$. The change-point model has the form given by

$$\text{logit}\{F_{j,k}(\boldsymbol{\theta})\} = (\alpha + \beta u_j + \gamma v_k)I(\alpha + \beta u_j + \gamma v_k \leq w) + wI(\alpha + \beta u_j + \gamma v_k > w),$$

where $I(\cdot)$ denotes an indicator function. Let $\eta(x) = \text{logit}(x)$ for $0 < x < 1$. Suppose that $Y_n = 0$. We see how condition A is checked as follows.

Step 1. Let $\hat{\alpha}_n$ denote a posterior mean of $\alpha$ based on the first $n$ patients. For fixed values of $\beta$, $\gamma$ and $w$, we take $\boldsymbol{\phi} = (\alpha_1, \beta, \gamma, w)$ and $\boldsymbol{\psi} = (\alpha_2, \beta, \gamma, w)$ for any $\alpha_1$ and $\alpha_2$. Then, by the mean value theorem, we have $F_{j,k}(\boldsymbol{\phi}) - F_{j,k}(\boldsymbol{\psi}) = \big[\eta\{F_{j,k}(\boldsymbol{\phi})\} - \eta\{F_{j,k}(\boldsymbol{\psi})\}\big]/\eta'(\tilde{F})$, where $\tilde{F}$ lies between $F_{j,k}(\boldsymbol{\phi})$ and $F_{j,k}(\boldsymbol{\psi})$, and $\eta'(F)$ denotes the derivative of $\eta$ with respect to $F$. We notice that $\eta'(x) = 1/\{x(1-x)\} \geq 0$ for $0 < x < 1$. For $\alpha_1 \leq \alpha_2$, there are three possible cases: (1) $\alpha_1 + \beta u_j + \gamma v_k \leq w$ and $\alpha_2 + \beta u_j + \gamma v_k \leq w$; (2) $\alpha_1 + \beta u_j + \gamma v_k \leq w$ and $\alpha_2 + \beta u_j + \gamma v_k > w$; and (3) $\alpha_1 + \beta u_j + \gamma v_k > w$ implying $\alpha_2 + \beta u_j + \gamma v_k > w$. If $\alpha_1 + \beta u_j + \gamma v_k \leq w$ and $\alpha_2 + \beta u_j + \gamma v_k \leq w$, then $\eta\{F_{j,k}(\boldsymbol{\phi})\} - \eta\{F_{j,k}(\boldsymbol{\psi})\} = \alpha_1 - \alpha_2 \leq 0$. This implies that $F$ is nondecreasing in $\alpha$. If $\alpha_1 + \beta u_j + \gamma v_k \leq w$ and $\alpha_2 + \beta u_j + \gamma v_k > w$, then $\eta\{F_{j,k}(\boldsymbol{\phi})\} - \eta\{F_{j,k}(\boldsymbol{\psi})\} = \alpha_1 + \beta u_j + \gamma v_k - w \leq 0$, which implies that $F$ is nondecreasing in $\alpha$. If $\alpha_1 + \beta u_j + \gamma v_k > w$, then $\eta\{F_{j,k}(\boldsymbol{\phi})\} - \eta\{F_{j,k}(\boldsymbol{\psi})\} = w - w = 0$, which implies that $F$ is constant in $\alpha$. Thus, for all three possible cases, $F$ is nondecreasing in $\alpha$. Thus, we have $(\alpha_1 - \alpha_2)\{F_{j,k}(\boldsymbol{\psi}) - F_{j,k}(\boldsymbol{\phi})\} \leq 0$ for any $\alpha_1$ and $\alpha_2$. This implies that $\hat{\alpha}_n \leq \hat{\alpha}_{n-1}$ and $(\hat{\alpha}_n - \hat{\alpha}_{n-1})\partial F/\partial \alpha \leq 0$.

Step 2. Let $\hat{\beta}_n$ denote the posterior mean of $\beta$ obtained from the first $n$ patients. Similar to the first step, for $\beta_1 \leq \beta_2$, we consider all possible cases for $\eta\{F_{j,k}(\boldsymbol{\phi})\} - \eta\{F_{j,k}(\boldsymbol{\psi})\}$: (1) $\alpha + \beta_1 u_j + \gamma v_k \leq w$ and $\alpha + \beta_2 u_j + \gamma v_k \leq w$; (2) $\alpha + \beta_1 u_j + \gamma v_k \leq w$ and $\alpha + \beta_2 u_j + \gamma v_k > w$; (3) $\alpha + \beta_1 u_j + \gamma v_k > w$ and $\alpha + \beta_2 u_j + \gamma v_k \leq w$; and (4) $\alpha + \beta_1 u_j + \gamma v_k > w$ and $\alpha + \beta_2 u_j + \gamma v_k > w$. Without loss of generality, we suppose that $\alpha + \beta_1 u_j + \gamma v_k \leq w$ and $\alpha + \beta_2 u_j + \gamma v_k \leq w$. Then, $\eta\{F_{j,k}(\boldsymbol{\phi})\} - \eta\{F_{j,k}(\boldsymbol{\psi})\} = (\beta_1 - \beta_2)u_j$. If $u_j \geq 0$, then $\eta\{F_{j,k}(\boldsymbol{\phi})\} - \eta\{F_{j,k}(\boldsymbol{\psi})\} \leq 0$, and $F$ is nondecreasing. This implies that $(\beta_1 - \beta_2)\{F_{j,k}(\boldsymbol{\psi}) - F_{j,k}(\boldsymbol{\phi})\} \leq 0$ for any $\beta_1$ and $\beta_2$ and $\hat{\beta}_n \leq \hat{\beta}_{n-1}$. If

$u_j < 0$, then $\eta\{F_{j,k}(\boldsymbol{\phi})\} - \eta\{F_{j,k}(\boldsymbol{\psi})\} \geq 0$, and $F$ is nonincreasing. This implies that $(\beta_1 - \beta_2)\{F_{j,k}(\boldsymbol{\psi}) - F_{j,k}(\boldsymbol{\phi})\} \geq 0$ for any $\beta_1$ and $\beta_2$ and $\hat{\beta}_n \geq \hat{\beta}_{n-1}$. Thus, regardless of the sign of the first agent $u_j$, we have $(\hat{\beta}_n - \hat{\beta}_{n-1})\partial F/\partial\beta \leq 0$.

Step 3. Similar to the second step, we can show that $(\hat{\gamma}_n - \hat{\gamma}_{n-1})\partial F/\partial\gamma \leq 0$.

Step 4. Let $\hat{w}_n$ denote the posterior mean of $w$ based on the first $n$ patients. For $w_1 \leq w_2$, we consider three possible cases: (1) $\alpha+\beta u_j+\gamma v_k \leq w_1(\leq w_2)$; (2) $w_1 < \alpha+\beta u_j+\gamma v_k \leq w_2$; and (3) $\alpha + \beta u_j + \gamma v_k > w_2(\geq w_1)$. If $\alpha + \beta u_j + \gamma v_k \leq w_1(\leq w_2)$, then $\eta\{F_{j,k}(\boldsymbol{\phi})\} - \eta\{F_{j,k}(\boldsymbol{\psi})\} = 0$. If $w_1 < \alpha+\beta u_j+\gamma v_k \leq w_2$, then $\eta\{F_{j,k}(\boldsymbol{\phi})\} - \eta\{F_{j,k}(\boldsymbol{\psi})\} = w_1 - (\alpha + \beta u_j + \gamma v_k) < 0$. If $\alpha+\beta u_j+\gamma v_k > w_2(\geq w_1)$, then $\eta\{F_{j,k}(\boldsymbol{\phi})\} - \eta\{F_{j,k}(\boldsymbol{\psi})\} = w_1 - w_2 \leq 0$. From all three cases, $F$ is nondecreasing in $w$ and $(w_1 - w_2)\{F_{i,k}(\boldsymbol{\psi}) - F_{j,k}(\boldsymbol{\phi})\} \leq 0$ for any $w_1$ and $w_2$. This implies that $\hat{w}_n \leq \hat{w}_{n-1}$ and $(\hat{w}_n - \hat{w}_{n-1})\partial F/\partial w \leq 0$.

Therefore, the inequality (3) holds when $Y_n = 0$. We can similarly show that the inequality (3) holds when $Y_n = 1$. □

**Example 3. Copula-type regression models** Yin and Yuan (2009) proposed two Copula-type regression models for drug combination trials: the Clayton-copula regression model and the Gumbel model. We first consider the Clayton-copula regression model, given by

$$F_{j,k}(\boldsymbol{\theta}) = 1 - \left\{(1 - p_j^{\alpha})^{-\gamma} + (1 - q_k^{\beta})^{-\gamma} - 1\right\}^{-1/\gamma},$$

where $\boldsymbol{\theta} = (\alpha, \beta, \gamma)$ with $\alpha, \beta, \gamma > 0$, and $p_j^{\alpha}$ and $q_k^{\beta}$ denoting the marginal true toxicity probability of level $j$ in agent 1 and level $k$ of agent 2, respectively. Let $G(x) = (1 - x)^{-\gamma}$. Then, $G\{F_{j,k}(\boldsymbol{\theta})\} = (1 - p_j^{\alpha})^{-\gamma} + (1 - q_k^{\beta})^{-\gamma} - 1$. Suppose that $Y_n = 0$. In what follows, we show that condition A holds when $Y_n = 0$.

Step 1. We consider the inequality (3) with respect to $\alpha$. For fixed values of $\beta$ and $\gamma$, we take

$\boldsymbol{\phi} = (\alpha_1, \beta, \gamma)$ and $\boldsymbol{\psi} = (\alpha_2, \beta, \gamma)$ for any $\alpha_1$ and $\alpha_2$. By the mean value theorem, we have $F_{j,k}(\boldsymbol{\phi}) - F_{j,k}(\boldsymbol{\psi}) = \big\{(1-p_j^{\alpha_1})^{-\gamma} - (1-p_j^{\alpha_2})^{-\gamma}\big\}/G'(\tilde{F})$ for some $\tilde{F}$ that lies between $F_{j,k}(\boldsymbol{\phi})$ and $F_{j,k}(\boldsymbol{\psi})$, where $G'(F)$ denotes the derivative of $G$ with respect to $F$. Since $G(x)$ is increasing and $g(x) = (1-p^x)^{-\gamma}$ for some $p \in (0,1)$ is decreasing, $F_{j,k}(\boldsymbol{\phi}) - F_{j,k}(\boldsymbol{\psi}) \geq 0$ for $\alpha_1 \leq \alpha_2$, i.e., $F$ is nonincreasing in $\alpha$. Also, $(\alpha_1 - \alpha_2)\{F_{j,k}(\boldsymbol{\psi}) - F_{j,k}(\boldsymbol{\phi})\} \geq 0$ for any $\alpha_1$ and $\alpha_2$. Let $\hat{\alpha}_n$ be a posterior mean of $\alpha$ based on the first $n$ patients. By using the approach used in the proof of Lemma 2, we have $\hat{\alpha}_n \geq \hat{\alpha}_{n-1}$. Thus, we obtain $(\hat{\alpha}_n - \hat{\alpha}_{n-1})\partial F/\partial \alpha \leq 0$.

Step 2. Similar to the first step, we can show that $F_{j,k}(\boldsymbol{\phi}) - F_{j,k}(\boldsymbol{\psi}) \geq 0$ for $\beta_1 \leq \beta_2$, where $\boldsymbol{\phi} = (\alpha, \beta_1, \gamma)$ and $\boldsymbol{\psi} = (\alpha, \beta_2, \gamma)$ for a fixed value of $\alpha$ and $\gamma$, and $\hat{\beta}_n \geq \hat{\beta}_{n-1}$, where $\hat{\beta}_n$ denotes the posterior mean of $\beta$ based on the first $n$ observations. This implies that $(\hat{\beta}_n - \hat{\beta}_{n-1})\partial F/\partial \beta \leq 0$.

Step 3. We check the inequality (3) with respect to $\gamma$. For fixed values of $\alpha$ and $\beta$, we consider $\boldsymbol{\phi} = (\alpha, \beta, \gamma_1)$ and $\boldsymbol{\psi} = (\alpha, \beta, \gamma_2)$ for any $\gamma_1$ and $\gamma_2$. Then,

$$F_{j,k}(\boldsymbol{\phi}) - F_{j,k}(\boldsymbol{\psi}) = \big\{(1-p_j^{\alpha})^{-\gamma_1} + (1-q_k^{\beta})^{-\gamma_1} - (1-p_j^{\alpha})^{-\gamma_2} - (1-q_k^{\beta})^{-\gamma_2}\big\}/G'(\tilde{F})$$

for some $\tilde{F}$ that lies between $F_{j,k}(\boldsymbol{\phi})$ and $F_{j,k}(\boldsymbol{\psi})$. Let $h(x) = (1-p^{\alpha})^{-x} + (1-q^{\beta})^{-x}$ for some $p, q \in (0,1)$. Then, $h(x)$ is increasing. Since $G(x)$ is also increasing, $F_{j,k}(\boldsymbol{\phi}) - F_{j,k}(\boldsymbol{\psi}) \leq 0$ for $\gamma_1 \leq \gamma_2$, i.e., $F$ is nondecreasing in $\gamma$. Also, we have $(\gamma_1 - \gamma_2)\{F_{j,k}(\boldsymbol{\psi}) - F_{j,k}(\boldsymbol{\phi})\} \leq 0$ for any $\gamma_1$ and $\gamma_2$, which implies that $\hat{\gamma}_n \leq \hat{\gamma}_{n-1}$, where $\hat{\gamma}_n$ denotes the posterior mean of $\gamma$ based on the first $n$ observations. So, we obtain $(\hat{\gamma}_n - \hat{\gamma}_{n-1})\partial F/\partial \gamma \leq 0$.

Therefore, the inequality (3) holds when $Y_n = 0$, and we can similarly show that the inequality (3) holds when $Y_n = 1$.

Next, we investigate the coherence of the Gumbel model, given by

$$F_{j,k}(\boldsymbol{\theta}) = 1 - (1-p_j^\alpha)(1-q_k^\beta)\big\{1 + p_j^\alpha q_k^\beta - 2p_j^\alpha q_k^\beta (e^\gamma+1)^{-1}\big\},$$

where $\boldsymbol{\theta} = (\alpha, \beta, \gamma)$ with $\alpha, \beta, \gamma > 0$, and $p_j^\alpha$ and $q_k^\beta$ denote the marginal true toxicity probability of level $j$ for agent 1 and level $k$ for agent 2, respectively. Let $G(x) = (1-x)/\big\{(1-p^\alpha)(1-q^\beta)\big\}$ for some $p, q \in (0,1)$. Then, $G\big\{F_{j,k}(\boldsymbol{\theta})\big\} = 1 + p_j^\alpha q_k^\beta - 2p_j^\alpha q_k^\beta/(e^\gamma+1)$. In what follows, we show that condition A holds when $Y_n = 0$.

Step 1. For fixed values of $\beta$ and $\gamma$, we first take $\boldsymbol{\phi} = (\alpha_1, \beta, \gamma)$ and $\boldsymbol{\psi} = (\alpha_2, \beta, \gamma)$ for any $\alpha_1$ and $\alpha_2$. By the mean value theorem, we have $F_{j,k}(\boldsymbol{\phi}) - F_{j,k}(\boldsymbol{\psi}) = (p_j^{\alpha_1} - p_j^{\alpha_2})\big\{q_k^\beta - 2q_k^\beta(e^\gamma+1)^{-1}\big\}/G'(\tilde{F})$ for some $\tilde{F}$ that lies between $F_{j,k}(\boldsymbol{\phi})$ and $F_{j,k}(\boldsymbol{\psi})$, where $G'(F)$ denotes the derivative of $G$ with respect to $F$. Since $(e^\gamma+1)^{-1} < 1/2$ for $\gamma > 0$ and $G$ is decreasing, we have $F_{j,k}(\boldsymbol{\phi}) - F_{j,k}(\boldsymbol{\psi}) \le 0$ for $\alpha_1 \le \alpha_2$, i.e., $F$ is nondecreasing in $\alpha$. Also, $(\alpha_1 - \alpha_2)\{F_{j,k}(\boldsymbol{\psi}) - F_{j,k}(\boldsymbol{\phi})\} \le 0$ for any $\alpha_1$ and $\alpha_2$. Thus, by using the approach used in the proof of Lemma 2, we have $\hat{\alpha}_n \le \hat{\alpha}_{n-1}$. Thus, we obtain $(\hat{\alpha}_n - \hat{\alpha}_{n-1})\partial F/\partial\alpha \le 0$.

Step 2. Similar to the first step, we can obtain $F_{j,k}(\boldsymbol{\phi}) - F_{j,k}(\boldsymbol{\psi}) \le 0$ for $\beta_1 \le \beta_2$, where $\boldsymbol{\phi} = (\alpha, \beta_1, \gamma)$ and $\boldsymbol{\psi} = (\alpha, \beta_2, \gamma)$ for fixed values of $\alpha$ and $\gamma$, and $(\beta_1 - \beta_2)\{F_{j,k}(\boldsymbol{\psi}) - F_{j,k}(\boldsymbol{\phi})\} \le 0$ for any $\beta_1$and $\beta_2$, which implies that $\hat{\beta}_n \le \hat{\beta}_{n-1}$. Thus, $(\hat{\beta}_n - \hat{\beta}_{n-1})\partial F/\partial\beta \le 0$.

Step 3. For fixed values of $\alpha$ and $\gamma$, we take $\boldsymbol{\phi} = (\alpha, \beta, \gamma_1)$ and $\boldsymbol{\psi} = (\alpha, \beta, \gamma_2)$ for any $\gamma_1$ and $\gamma_2$. Then, $F_{j,k}(\boldsymbol{\phi}) - F_{j,k}(\boldsymbol{\psi}) = 2p_i^\alpha q_k^\beta\big\{(e^{\gamma_2}+1)^{-1} - (e^{\gamma_1}+1)^{-1}\big\}/G'(\tilde{F})$ for some $\tilde{F}$ that lies between $F_{j,k}(\boldsymbol{\phi})$ and $F_{j,k}(\boldsymbol{\psi})$. Since $G(x)$ is decreasing and $g(x) = (e^x+1)^{-1}$ is decreasing, $F_{j,k}(\boldsymbol{\phi}) - F_{j,k}(\boldsymbol{\psi}) \ge 0$ for $\gamma_1 \le \gamma_2$ and $(\gamma_1 - \gamma_2)\{F_{j,k}(\boldsymbol{\psi}) - F_{j,k}(\boldsymbol{\phi})\} \ge 0$ for any $\gamma_1$and $\gamma_2$ implying $\hat{\gamma}_n \ge \hat{\gamma}_{n-1}$. So, $(\hat{\gamma}_n - \hat{\gamma}_{n-1})\partial F/\partial\gamma \le 0$.

Therefore, the inequality (3) holds when $Y_n = 0$. We can similarly show that the inequality (3) holds when $Y_n = 1$. □

**Example 4. Log-linear model** Wang and Ivanova (2005) considered a toxicity model given by

$$\log\{1 - F_{j,k}(\boldsymbol{\theta})\} = \alpha \log(1 - u_j) + \beta \log(1 - v_k) + \gamma \log(1 - u_j)\log(1 - v_k), \qquad (1)$$

with $\boldsymbol{\theta} = (\alpha, \beta, \gamma)$, where $\alpha > 0, \beta > 0$ and $\gamma < 0$. Let $G_{j,k}(\boldsymbol{\theta}) = \alpha \log(1 - u_j) + \beta \log(1 - v_k) + \gamma \log(1 - u_j)\log(1 - v_k)$. Then, $F_{j,k}(\boldsymbol{\theta}) = 1 - \exp\{G_{j,k}(\boldsymbol{\theta})\}$. Suppose that $Y_n = 0$. In what follows, we show that the design with the model in (1) is coherent by checking condition A.

Step 1. Let $\hat{\alpha}_n$ denote a posterior mean of $\alpha$ based on the first $n$ patients. For fixed values of $\beta$ and $\gamma$, we take $\boldsymbol{\phi} = (\alpha_1, \beta, \gamma)$ and $\boldsymbol{\psi} = (\alpha_2, \beta, \gamma)$ for any $\alpha_1$ and $\alpha_2$. Then, $F_{j,k}(\boldsymbol{\phi}) - F_{j,k}(\boldsymbol{\psi}) = \{G_{j,k}(\boldsymbol{\psi}) - G_{j,k}(\boldsymbol{\phi})\}\exp(\tilde{G})$ for some $\tilde{G}$ that lies between $G_{j,k}(\boldsymbol{\phi})$ and $G_{j,k}(\boldsymbol{\psi})$. And $G_{j,k}(\boldsymbol{\psi}) - G_{j,k}(\boldsymbol{\phi}) = (\alpha_2 - \alpha_1)\log(1 - u_j)$. If $u_j < 0$, then $G_{j,k}(\boldsymbol{\psi}) - G_{j,k}(\boldsymbol{\phi}) \geq 0$ for $\alpha_1 \leq \alpha_2$, which implies that $F_{j,k}(\boldsymbol{\phi}) - F_{j,k}(\boldsymbol{\psi}) \geq 0$ for $\alpha_1 \leq \alpha_2$, i.e., $F$ is nondecreasing in $\alpha$. This yields $(\alpha_1 - \alpha_2)\{F_{j,k}(\boldsymbol{\psi}) - F_{j,k}(\boldsymbol{\phi})\} \geq 0$ for any $\alpha_1$ and $\alpha_2$, and by the approach used in the proof of Lemma 2, $\hat{\alpha}_n \geq \hat{\alpha}_{n-1}$. So, $(\hat{\alpha}_n - \hat{\alpha}_{n-1})\partial F/\partial\alpha \leq 0$. Likewise, we obtain $(\hat{\alpha}_n - \hat{\alpha}_{n-1})\partial F/\partial\alpha \leq 0$ even if $u_j \geq 0$. In other words, regardless of the sign of the dose for the first agent $u_j$, we obtain $(\hat{\alpha}_n - \hat{\alpha}_{n-1})\partial F/\partial\alpha \leq 0$.

Step 2. Similar to the first step, we can show that $(\hat{\beta}_n - \hat{\beta}_{n-1})\partial F/\partial\beta \leq 0$.

Step 3. For fixed values of $\alpha$ and $\beta$, we take $\boldsymbol{\phi} = (\alpha, \beta, \gamma_1)$ and $\boldsymbol{\psi} = (\alpha, \beta, \gamma_2)$ for any $\gamma_1$ and $\gamma_2$. Then, $G_{j,k}(\boldsymbol{\psi}) - G_{j,k}(\boldsymbol{\phi}) = (\gamma_2 - \gamma_1)\log(1 - u_j)\log(1 - v_k)$. According to the sign of doses $u_j$ and $v_k$, $G$ is nonincreasing or nondecreasing in $\gamma$. Suppose that $u_j \geq 0$ and

$v_k < 0$. Then, $\log(1-u_j) \leq 0$ and $\log(1-v_k) > 0$. So, $G_{j,k}(\boldsymbol{\psi}) - G_{j,k}(\boldsymbol{\phi}) \leq 0$ for $\gamma_1 \leq \gamma_2$ and $F$ is nondecreasing in $\gamma$. Thus, $(\gamma_1 - \gamma_2)\{F_{j,k}(\boldsymbol{\psi}) - F_{j,k}(\boldsymbol{\phi})\} \leq 0$ for any $\gamma_1$ and $\gamma_2$. By using the approach used in the proof of Lemma 2, we obtain $(\hat{\gamma}_n - \hat{\gamma}_{n-1})\partial F/\partial\gamma \leq 0$, where $\hat{\gamma}_n$ denotes the posterior mean of $\gamma$ based on the first $n$ patients. Similar to the case where $u_j \geq 0$ and $v_k < 0$, we can check $(\hat{\gamma}_n - \hat{\gamma}_{n-1})\partial F/\partial\gamma \leq 0$ regardless of the sign of the doses of the two agents.

Therefore, the inequality (3) holds when $Y_n = 0$. We can similarly show that the inequality (3) holds when $Y_n = 1$. □

**Example 5. Six-parameter model** Thall et al. (2003) proposed a six-parameter drug combination toxicity model, given by

$$F_{j,k}(\boldsymbol{\theta}) = \frac{\alpha_1 u_j^{\beta_1} + \alpha_2 v_k^{\beta_2} + \alpha_3\left(u_j^{\beta_1} v_k^{\beta_2}\right)^{\beta_3}}{1 + \alpha_1 u_j^{\beta_1} + \alpha_2 v_k^{\beta_2} + \alpha_3\left(u_j^{\beta_1} v_k^{\beta_2}\right)^{\beta_3}},$$

where $\boldsymbol{\theta} = (\alpha_1, \alpha_2, \alpha_3, \beta_1, \beta_2, \beta_3)$. The doses are standardized such that $0 \leq u_j \leq 1$ and $0 \leq v_k \leq 1$. To ensure that the marginal toxicity probability is increasing with the dose, we require $\alpha_1 > 0, \alpha_2 > 0$, $\alpha_3\left(v_k^{\beta_2}\right)^{\beta_3} > 0$, $k = 1, \ldots, K$, and $\alpha_3\left(u_j^{\beta_1}\right)^{\beta_3} > 0$, $j = 1, \ldots, J$. Let $G_{j,k}(\boldsymbol{\theta}) = \alpha_1 u_j^{\beta_1} + \alpha_2 v_k^{\beta_2} + \alpha_3\left(u_j^{\beta_1} v_k^{\beta_2}\right)^{\beta_3}$. Then, $F_{j,k}(\boldsymbol{\theta}) = G_{j,k}(\boldsymbol{\theta})/\{1 + G_{j,k}(\boldsymbol{\theta})\}$. Suppose that $Y_n = 0$. We easily see that $\partial G/\partial\alpha_1 = u_j^{\beta_1} \geq 0$, $\partial G/\partial\alpha_2 = v_k^{\beta_2} \geq 0$ and $\partial G/\partial\alpha_3 = \left(u_j^{\beta_1} v_k^{\beta_2}\right)^{\beta_3} \geq 0$. So, $G$ is nondecreasing in $\alpha_1, \alpha_2$ and $\alpha_3$ and $F$ is nondecreasing in $\alpha_1, \alpha_2$ and $\alpha_3$, because $f(x) = x/(1+x)$ is nondecreasing in $x$. Then, for each $t = 1, 2, 3$, we obtain $(\alpha_{t1} - \alpha_{t2})\{F_{j,k}(\boldsymbol{\psi}_t) - F_{j,k}(\boldsymbol{\phi}_t)\} \leq 0$, where $\boldsymbol{\phi}_t$ replaces the $t$th element of $\boldsymbol{\theta}$ with $\alpha_{t1}$, and $\boldsymbol{\psi}_t$ replaces the $t$th element of $\boldsymbol{\theta}$ with $\alpha_{t2}$ for any $\alpha_{t1}$ and $\alpha_{t2}$. Also, we see that $\partial G/\partial\beta_1 = \alpha_1 u_j^{\beta_1} \log u_j + \alpha_3 v_k^{\beta_2\beta_3} u_j \log u_j \leq 0$, $\partial G/\partial\beta_2 = \alpha_2 v_k^{\beta_2} \log v_k + \alpha_3 u_j^{\beta_1\beta_3} v_k^{\beta_2} \log v_k \leq 0$ and $\partial G/\partial\beta_3 = \alpha_3(u_j^{\beta_1} v_k^{\beta_2})^{\beta_3} \log(u_j^{\beta_1} v_k^{\beta_2}) \leq 0$. This means that $G$ is nonincreasing in $\beta_1, \beta_2$ and $\beta_3$ and $F$ is nonincreasing in $\beta_1, \beta_2$ and $\beta_3$. Then, for each $t = 1, 2, 3$, we obtain

$(\beta_{t1}-\beta_{t2})\{F_{j,k}(\boldsymbol{\psi}_t)-F_{j,k}(\boldsymbol{\phi}_t)\} \leq 0$, where $\boldsymbol{\phi}_t$ replaces the $(t+3)$th element of $\boldsymbol{\theta}$ with $\beta_{t1}$ and $\boldsymbol{\psi}_t$ replaces the $(t+3)$th element of $\boldsymbol{\theta}$ with $\beta_{t2}$ for any $\beta_{t1}$ and $\beta_{t2}$. Let $\hat{\alpha}_{n,t}$ and $\hat{\beta}_{n,t}$, $t=1,2,3$ denote the posterior mean of $\alpha_t$ of $\beta_t$, respectively, based on the first $n$ patients. Then, by the approach used in the proof of Lemma 2, $\hat{\alpha}_{n,t} \leq \hat{\alpha}_{n-1,t}$ and $\hat{\beta}_{n,t} \geq \hat{\beta}_{n-1,t}$ for all $t=1,2,3$. Thus, $(\hat{\alpha}_{n,t}-\hat{\alpha}_{n-1,t})\partial F/\partial\alpha_t \leq 0$ and $(\hat{\beta}_{n,t}-\hat{\beta}_{n-1,t})\partial F/\partial\beta_t \leq 0$ for all $t=1,2,3$. Therefore, the inequality (3) holds when $Y_n=0$. We can similarly show that the inequality (3) holds when $Y_n=1$. □

# References


Braun, T. M. and Jia, N. (2013). A generalized continual reassessment method for two-agent phase I trials. *Statistics in biopharmaceutical research* **5** 105–115.

Thall, P. F., Millikan, R. E., Mueller, P. and Lee, S.-J. (2003). Dose-finding with two agents in phase I oncology trials. *Biometrics* **59** 487–496.

Wang, K. and Ivanova, A. (2005). Two-dimensional dose finding in discrete dose space. *Biometrics* **61** 217–222.

Yin, G. and Yuan, Y. (2009). Bayesian dose finding in oncology for drug combinations by copula regression. *Journal of the Royal Statistical Society: Series C (Applied Statistics)* **58** 211–224.
