# Supplementary material for: On the coherence of model-based dose-finding designs for drug combination trials
Source: PLoS One. 2020 Nov 30;15(11):e0242561. doi: 10.1371/journal.pone.0242561 (PMC7703981; doi:10.1371/journal.pone.0242561)
Supplement: Attachment — Submitted filename: response letter_v2.docx [file pone.0242561.s002.docx]

October 15, 2020

Dear Editors,

We are submitting a revised manuscript PONE-D-20-25012, “On the coherence of model-based dose-finding designs for drug combination trials.” We are grateful to you and the reviewers for careful review and comments. In the revision, we have revised manuscript carefully to address all concerns raised by reviewers. We hope you find the revision satisfactory.

Below, we provide point-by-point responses to all reviewers’ comments. For clarity, we include a copy of each reviewer’s comments in italics, followed by our response.

Sincerely,

Yeonhee Park, Ph.D.

Assistant Professor of Biostatistics

Department of Biostatistics and Medical Informatics

University of Wisconsin

**Point-by-point responses to comments from the reviewers:**

*Reviewer #1: This paper has been well written. From my point of view, defining a coherence conditionally to the scenario has no interest as the scenario is always unknown. However, the application of ND coherence may be useful to biostatistician working on the protocol of early phase dose finding study.*

*Reviewer #2: This is a very well written paper that describes the concept of coherence in drug combination trials and provides sufficient conditions for a model-based design to be coherent. As drug combinations studies are increasingly used in treating cancer, this paper adds an important element to the literature on the coherence of dose finding designs. I have a few very minor comments.*

1. *The authors considered some model-based designs in the literature. Could the authors briefly comment on if there are any commonly used designs in the literature that are not coherent?*

**Response:** Thank you for comment. One example is partial-order CRM (Wages et al. 2011), which fits multiple (single-agent) CRM models, each assuming a different toxicity order for the combinations, and then uses Bayesian model averaging (BMA) to summarize the estimates over different models and make the decision of dose transition. Although each CRM model is coherent, averaging over them would lead to incoherent decisions. This is because each of the CRM models may recommend a different dose-transition decision, and the final decision based on BMA would be consistent with some, but inconsistent with others, leading to incoherence. We have addressed this point in Discussion, Section 4.

2. *Could the authors briefly comment on how to deal with delayed toxicity?*

**Response:** Thank you for comment. Bayesian data augmentation can be adopted to handle the delayed toxicity and facilitate real-time decision making of dose escalation and de-escalation (Liu and Ning, 2013 and Liu et al. 2013). We have added comments on this issue to Discussion, Section 4.

Here are references mentioned above.

Liu S, Ning J. A Bayesian dose-finding design for drug combination trials with delayed toxicities. Bayesian analysis. 2013 Sep;8(3):703.

Liu S, Yin G, Yuan Y. Bayesian data augmentation dose finding with continual reassessment method and delayed toxicity. The annals of applied statistics. 2013 Dec 1;7(4):1837.

Wages NA, Conaway MR, O'Quigley J. Continual reassessment method for partial ordering. Biometrics. 2011 67(4):1555-63.
